# Altered functional connectivity related to prepulse inhibition in functional movement disorder^☆^

**DOI:** 10.1016/j.nicl.2026.103966

**Published:** 2026-02-15

**Authors:** David Voženílek, Karsten Mueller, Tereza Serranová, Lucia Nováková, Manuela Vaněčková, Petr Sojka

**Affiliations:** a Department of Psychiatry, Faculty of Medicine, Masaryk University, Brno, Czech Republic; b Central European Institute of Technology, Masaryk University, Brno, Czech Republic; c Max Planck Institute for Human Cognitive and Brain Sciences, Leipzig, Germany; d Department of Neurology, Charles University, 1st Faculty of Medicine and General University Hospital, Prague, Czech Republic; e Department of Radiology, Charles University, 1st Faculty of Medicine and General University Hospital, Prague, Czech Republic

**Keywords:** Functional Neurological Disorder, Prepulse Inhibition, Sensorimotor Gating, Functional Connectivity, Temporoparietal Junction, Ventral attention network

## Abstract

**Background:**

Functional neurological disorder (FND) is increasingly understood as a multinetwork disorder. Deficits in subcortical sensorimotor gating, indexed by reduced prepulse inhibition (PPI) of the startle reflex, are reported in FND and may reflect impaired filtering and contextual weighting of sensory input. However, the neural correlates linking impaired gating to intrinsic network dysfunction remain unclear.

**Methods:**

We examined the relationship between PPI and resting-state functional connectivity in 38 patients with functional movement disorder (FMD) and 39 healthy controls (HC). PPI of the blink reflex was assessed using a standardized neurophysiological paradigm, and seed-based connectivity (SBC) analyses were performed in CONN toolbox using the default atlas comprising cortical and subcortical regions across the whole brain. To investigate a potential interaction between group and PPI size, we tested whether associations between PPI and SBC differed between FMD and HC groups.

**Results:**

FMD patients exhibited significantly reduced PPI compared to HCs. Across several seeds—the left insular cortex, right supramarginal gyrus and bilateral anterior insula—significant Group × PPI interactions emerged in bilateral temporoparietal junction (TPJ). In HCs, higher PPI was associated with stronger connectivity between the insula and TPJ and between bilateral TPJ regions. These relationships were absent in FMD patients.

**Conclusions:**

Our findings demonstrate that effective sensorimotor gating depends on intact functional coupling within insula–TPJ circuits, key nodes of the salience and ventral attention networks. Disrupted modulation of this network in FMD suggests impaired top-down filtering of sensory information, providing a mechanistic link between sensory processing deficits and aberrant salience-network dynamics in FMD.

## Highlights

• FMD patients showed reduced prepulse inhibition compared to healthy controls.
• PPI correlates with insula-TPJ connectivity in controls but not in FMD patients.
• Disrupted salience attribution may relate to impaired sensory filtering in FMD.

## 1 Introduction

Functional Neurological Disorder (FND) represents a prevalent and disabling group of conditions, in which patients experience genuine neurological symptoms, such as abnormal movements, sensory disturbances, or seizure-like episodes that are characterized by internal inconsistency (Hallett et al., 2022). Increasing evidence highlights that FND arises from complex interactions among biological, psychological, and social factors, manifesting as disruptions in brain function (Edwards et al., 2012, Ludwig et al., 2018, Drane et al., 2020). Neuroimaging studies have demonstrated abnormalities in large-scale networks, including altered functional connectivity within and between the salience, limbic, and frontoparietal attention networks, alongside disruptions in brain regions critical for the sense of agency and the integration of sensory information (Perez et al., 2021).

A growing body of evidence suggests that altered processing of sensory information is an important feature of FND. Patients exhibit abnormalities across multiple sensory modalities, including impaired temporal discrimination and altered responses to somatosensory, interoceptive, or nociceptive stimuli (Tinazzi et al., 2014, Matthews et al., 2020, Sadnicka et al., 2020, Squintani et al., 2024, Stoffel et al., 2025). These findings point toward disruptions in mechanisms that regulate how sensory inputs are filtered and integrated − processes that can be directly indexed using prepulse inhibition (PPI) of the startle reflex, a robust and well-characterized measure of sensorimotor gating (Blumenthal and Gescheider, 1987). PPI occurs when a sensory stimulus (the prepulse) too weak to trigger a reflex on its own, reduces the intensity of a reflex response to a stronger stimulus presented 30 to 500 ms later. PPI is widely accepted as a key measure of sensorimotor gating, a physiological mechanism that regulates sensory input by integration of competing stimuli.

PPI reflects early, pre-attentive filtering of sensory information that protects ongoing processing from disruption (Swerdlow, Geyer and Braff, 2001). Its magnitude is shaped by stimulus salience and indexes automatic selection processes occurring before conscious awareness (Filion et al., 1993, Corbetta et al., 2008, Sara and Bouret, 2012, Correa et al., 2019, Kofler et al., 2024). When PPI is reduced, low-salience stimuli are insufficiently integrated or filtered, leading to decreased sensory fidelity and greater perceptual noise. Impaired PPI has been reported across numerous neuropsychiatric and somatic symptom conditions possibly reflecting deficits in early sensory gating at subcortical level as well as in the forebrain mechanisms that modulate inhibition of irrelevant sensory input (Swerdlow et al., 2016, Santos-Carrasco and De la Casa, 2023). Despite the widespread PPI impairment, only limited or inconsistent links have been found between PPI and clinical measures of severity in disorders such as schizophrenia (San-Martin et al., 2020), suggesting that PPI may relate to a more general vulnerability, rather than to specific symptoms. In FND, reduced PPI has been observed in both functional motor and functional seizure subtypes, and similar abnormalities have been noted in related disorders with prominent pain such as fibromyalgia and interstitial cystitis (Pouretemad et al., 1998, Kilpatrick et al., 2010, Kofler and Halder, 2014, Hanzlíková et al., 2019). These disruptions likely arise from heterogeneous mechanisms, including genetic vulnerability and neural circuit dysfunction due to a disease (Light and Swerdlow, 2014, Swerdlow et al., 2016). Accordingly, PPI is increasingly viewed as a transdiagnostic marker of impaired sensorimotor gating.

Recent findings in functional movement disorder (FMD), one of the most common subtypes of FND (Hallett et al., 2022), further showed that reduced PPI was closely associated with patients’ subjective symptom burden and widespread pain, whereas it did not correlate with clinician-rated motor severity, suggesting that disrupted sensory gating at the subcortical level may contribute primarily to symptom experience than motor dysfunction (Nováková et al., 2025). PPI is regulated by top-down forebrain projections including the cortico-striatal-pallidal-thalamic network that modulates brainstem startle circuitry (Naysmith, Kumari and Williams, 2021). Notably, many of the regions that modulate PPI are the same regions repeatedly implicated in neuroimaging studies in FND, which is viewed as a multinetwork disorder (Drane et al., 2020), including of the salience and ventral attention networks responsible for detecting, prioritizing, and integrating interoceptive and exteroceptive stimuli (Diez et al., 2019).

Converging lines of evidence indicate that FMD is characterized by both disrupted sensory processing, including deficits in early sensory gating, and abnormal connectivity within large-scale brain networks implicated in sensorimotor integration and salience detection. However, these two domains of dysfunction have largely been investigated in isolation. Bridging these findings, the present study assessed PPI, a marker of sensorimotor gating, together with resting-state functional magnetic resonance imaging (rs-fMRI) to investigate the relationship between PPI and functional connectivity in a cohort of 39 patients with FMD and 40 healthy controls (HCs). In particular, we aim to establish whether the relationship between functional connectivity and PPI size is different in FMD patients and healthy controls within the networks and areas associated with PPI or FMD.

## 2 Methods

### 2.1 Participants

Seventy-nine subjects were recruited between May 2020 and May 2023 and gave a written consent to take part in this study. Participants were excluded if they were under 18 years old or had a history of severe learning disabilities, cognitive impairment, or language difficulties. Additional exclusion criteria included a history of organic neurological brain disorders, substance dependence, psychosis, significant illnesses associated with non-motor symptoms, or medication known to affect PPI (such as dopamine receptor antagonists). The study was approved by the Ethics Committee of the General Teaching Hospital in Prague, approval number: 37/19 Grant AZV VES 2020 1. LF UK.

FMD patients were recruited at the specialized outpatient service for FMD at the Neurology Department, 1st Faculty of Medicine and General University Hospital in Prague.

HCs were matched to the FMD patients based on age and sex. They were recruited using a directory of healthy volunteers interested in participation in clinical studies, operated by the Neurology Department. Controls went through a screening process, which included taking a complete medical history and performing a full neurological examination, to exclude any that suffer from sensorimotor symptoms or objective signs of neurological disorders. All HCs received financial compensation of $25.

Participants from both groups underwent a full neurological assessment performed by a neurologist with experience in FMD diagnosis. The examination, including a thorough clinical interview, focused on positive signs of functional weakness or movements that were abnormal, as well as inconsistent and incongruent with other movement disorders. The clinically definite FMD diagnosis was based on Gupta and Lang criteria. Primary symptoms of FMD patients were categorized as weakness (12; 31.6%), gait abnormalities (12; 31.6%), functional tremor (9; 23.7%), dystonia (4; 10.5%) or myoclonus (1; 2.6%).

As part of the assessment, a detailed pharmacological history of each participant, as well as current medication use were recorded and it was ensured that no participant used PPI-affecting medication, for example dopamine receptor antagonists. During a structured interview, medical comorbidities, family history, the use of drugs, caffeine, tobacco products were assessed. Prior to PPI testing, participants were instructed to avoid smoking and caffeine consumption, because of known effects of caffeine and nicotine on sensorimotor gating. The handedness of each patient was also recorded.

All participants completed a range of clinical measures and questionnaires.

### 2.2 Questionnaires

Depressive symptoms were assessed using the Beck Depression Inventory (BDI-II), a 21 item self-report inventory. Each item is scored on a scale of 0 to 3, with scores being summed for a total score of 0 to 63.

State and trait anxiety was assessed using the State-Trait Anxiety Inventory (STAI). It consists of two sets of 20 questions, with one measuring state anxiety and the other trait anxiety. Each question is rated by the participant on a 4-point frequency scale, leading to a state and trait anxiety score of 20 to 80.

In patients, severity of motor disorders was assessed using the Simplified FMD Rating Scale (S-FMDRS). The severity and duration of abnormal movements across seven body regions were rated based on a recording of the patient, resulting in a total score with a maximum of 54.

Somatic symptoms were measured with the modified Patient Health Questionnaire (F-PHQ) (Carson et al., 2015), which includes the PHQ-15 items (excluding menstrual and sexual symptoms), additional neurological and mental state items, and specific items assessing tremor/jerks and abnormal posture/spasms. Symptoms are rated by the participant on a scale of 0 to 2, resulting in a total score of 0 to 54. Subjective Motor Symptom Severity (SMSS) was derived from a five-item subset of the F-PHQ, with a final score of 0 to 10 (Forejtová et al., 2023).

Fatigue was evaluated with the Fatigue Severity Scale (FSS) (Krupp et al., 1989), a 9-item scale with the range 1–7, and pain with the PainDetect scale (Freynhagen et al., 2006) for current, average, and maximum pain in the previous 4 weeks; a composite score (0–30) was used for analysis.

### 2.3 Prepulse inhibition paradigm

#### 2.3.1 Neurophysiological examination

The neurophysiological assessment was performed under standardized conditions in an appropriately lit and quiet room. Participants were seated with the electromyographic recording apparatus positioned out of sight to minimize anticipatory responses. Prior to testing, participants received a detailed explanation of the various stimulus modalities. Data acquisition utilized a clinical-grade electrodiagnostic system (Synergy, CareFusion, London, UK) with band-pass filtering set between 30 and 30,000 Hz and a sampling rate of 2,000 Hz.

#### 2.3.2 Paradigm

Surface electromyographic activity of the orbicularis oculi was recorded using 10-mm gold electrodes and conductive gel. The active electrode was centered beneath the lower eyelid, bisecting the muscle, while the reference electrode was placed 2 cm laterally near the lateral canthus. Blink reflexes were evoked by delivering a 0.5-ms rectangular constant-current pulse to the right supraorbital nerve, with the cathode over the supraorbital incisura and the anode positioned 3 cm lateral along the nerve above the eyebrow.

Stimulus intensity for blink reflex elicitation was set at 10 times the individual’s sensory threshold, defined as the minimal current perceived in at least 4 of 8 trials. A conditioning prepulse of 0.2 ms in duration was applied to the right index finger (ring electrodes over proximal and middle phalanges) 100 ms before supraorbital stimulation, at twice the subject’s sensory threshold. Each participant underwent nine unconditioned (baseline) and nine conditioned (prepulse + reflex) stimulations, with interstimulus intervals of 8–10 s.

During both baseline and prepulse conditions, the waveform morphology (R1, R2, R3 components) was examined. Only subjects exhibiting no reduction or an increase in R1 amplitude in the prepulse condition were included in subsequent analyses. Subjective discomfort was quantified using a Numerical Rating Scale (0 = no discomfort, 10 = maximal tolerable pain).

#### 2.3.3 Analysis

All electromyographic records were rectified and processed offline. For each participant, 18 trials were analyzed: nine baseline and nine prepulse-conditioned. The ipsilateral early R1 and late R2 components, as well as the contralateral R2 (R2c), were identified in each recording. Reflex magnitudes were quantified by calculating the area under the curve (ms/mV) for R2 and R2c and then averaged. Although an R3 component was occasionally observed, it was excluded from quantitative analyses.

PPI was assessed by comparing mean reflex magnitudes between conditions. For each subject, mean areas for R2 and R2c were computed across nine trials per condition. To account for interindividual variability, reflex magnitudes in the prepulse condition (%PPI) were normalized to baseline magnitude (expressed as a percentage):

%PPI = (mean reflex magnitude_prepulse / mean reflex magnitude_baseline) × 100.

The primary outcome, PPI size, was defined as the percentage reduction from baseline:

PPI size = 100% – %PPI.

This metric reflects the extent of prepulse inhibition for each individual. All recorded EMG signals were of sufficient quality to permit comprehensive offline analysis.

### 2.4 MRI acquisition

Magnetic resonance imaging was performed on a 3  T Siemens MAGNETOM Skyra scanner equipped with a 32-channel head coil. High-resolution structural images were first acquired using a T1-weighted magnetization-prepared rapid gradient-echo (MPRAGE) (Mugler and Brookeman, 1991) sequence (TR/TE = 2300/2.9  ms, TI = 900  ms, flip angle = 9°, FOV = 256 × 240 × 212  mm, matrix = 256 × 240, voxel size = 1.0 × 1.0 × 1.2  mm).

Resting-state functional images were then obtained using a gradient-echo echo-planar imaging (EPI) sequence (TR/TE = 2000/30  ms, flip angle = 90°, FOV = 192 × 192  mm, matrix = 64 × 64, 30 axial slices, slice thickness = 3 mm, no gap, voxel size = 3 × 3 × 3 mm) with fat saturation. The slices were acquired in an interleaved ascending order, aligned 22.9° off the AC-PC line to minimize susceptibility artifacts. A total of 304 volumes were collected over approximately 10 min. During acquisition, participants were instructed to keep their eyes open and maintain fixation on a centrally presented crosshair.

### 2.5 MRI preprocessing

Analyses of fMRI data were performed using the functional connectivity toolbox CONN (Whitfield-Gabrieli and Nieto-Castanon, 2012) (RRID:SCR_009550) release 22.v2407 (Nieto-Castanon and Whitfield-Gabrieli, 2022) and SPM (Friston et al., 1994) (RRID:SCR_007037) release 12.7771.

Functional and anatomical data were processed using a modular CONN pipeline (Nieto-Castanon, 2020c), including realignment with susceptibility distortion correction (generating voxel-displacement maps using fieldmaps), slice-timing correction, outlier detection, segmentation, normalization to a standard space according to the template of the Montreal Neurological Institute (MNI), and spatial smoothing. Functional data were realigned using SPM’s *realign & unwarp* procedure (Andersson et al., 2001) with integrated fieldmaps to jointly correct for motion and susceptibility distortions via a 6-parameter rigid-body transformation and b-spline resampling (Friston et al., 1995, Andersson et al., 2001). Slice-timing correction employed sinc interpolation to resample slices to the mid-acquisition time (Henson et al., 1999, Sladky et al., 2011). Outlier scans were detected with Artifact detection tools (ART) (Whitfield-Gabrieli, Nieto-Castanon and Ghosh, 2011) as those with framewise displacement > 0.9 mm or global signal changes > 5 SD (Power et al., 2014, Nieto-Castanon, 2025); mean reference images were computed excluding these scans. Anatomical and functional data were coregistered, segmented (grey matter, white matter, cerebro-spinal fluid (CSF)), normalized to the MNI template, and resampled to 2 mm isotropic voxels using SPM’s unified segmentation and normalization algorithm (Ashburner and Friston, 2005, Ashburner, 2007, Calhoun et al., 2017, Nieto-Castanon, 2025) with the IXI-549 tissue probability map. Data were smoothed with a Gaussian kernel of 8 mm full width at half maximum (FWHM).

Functional data were denoised using the standard CONN pipeline (Nieto-Castanon, 2020a), regressing out five white matter and five CSF CompCor components (Behzadi et al., 2007, Chai et al., 2012), 12 motion parameters (six motion regressors and their first derivatives) (Friston et al., 1996), ART-identified outliers (Power et al., 2014), session effects and derivatives (2 regressors), and linear trends (2 regressors). Time series were bandpass filtered using the standard cutoff frequencies in CONN (0.008–0.09 Hz) (Hallquist, Hwang and Luna, 2013). CompCor components were extracted within eroded white matter and CSF masks as principal components orthogonal to mean signal, motion, and outlier effects. The effective post-denoising degrees of freedom ranged from 41.3 to 91.2 (mean = 87.8) across subjects (Nieto-Castanon, 2025).

### 2.6 Statistical analysis

#### 2.6.1 First-level analysis

Seed-based connectivity maps (SBC) were estimated characterizing the spatial pattern of functional connectivity with a seed area. Seed regions included 164 independent component analysis (ICA) based network templates provided by the CONN toolbox (Nieto-Castanon and Whitfield-Gabrieli, 2022) and Harvard–Oxford atlas regions of interest (ROIs) (Desikan et al., 2006). Functional connectivity strength was represented by Fisher-transformed bivariate correlation coefficients from a weighted general linear model (weighted general linear model (GLM) (Nieto-Castanon, 2020b)), estimated separately for each seed area and target voxel, modelling the association between their signal timeseries. To compensate for possible transient magnetization effects at the beginning of each run, individual scans were weighted by a step function convolved with an SPM canonical hemodynamic response function and rectified.

#### 2.6.2 Second-level analyses

Exploratory SBC analysis was performed on all seed regions of the atlas. The resulting first-level SBC maps for each of the selected seeds were entered into a GLM that included a Group (FMD, HC) × PPI interaction term to test whether the association between PPI and connectivity differed between groups, while controlling for age and sex as covariates of no interest. PPI and age covariates were mean-centered prior to model entry.

First, the main effect of the group was assessed by testing for differences in mean seed-based connectivity between FMD and HC patients. Second, we tested the Group × PPI interaction to identify regions where the linear relationship (slope) between SBC and PPI scores differed significantly between the groups. Finally, in regions showing a significant interaction, post-hoc contrasts were used to examine the PPI-connectivity relationship within each group individually to characterize the nature of the interaction.

All analyses were computed at the full-brain level, where seed-to-voxel parameter estimates were performed for all voxels within the entire brain for each seed region defined by the atlas. Voxel-level hypotheses regarding the interaction effects were evaluated using a general linear model with random-effects across subjects. Inferences were performed at the level of individual clusters. The resulting statistical parametric maps were initially assessed using a cluster-defining voxel threshold of *p* < 0.001 and a spatial threshold of *k* ≥ 300 voxels. To correct for multiple comparisons across the brain volume, significant clusters were identified using a family-wise error (FWE) correction with *p* < 0.05 at the cluster-level. Furthermore, to account for the number of parallel analyses conducted (one for each seed in the atlas), only results from seeds passing a Bonferroni corrected set-level threshold of *p* < 0.0003 are reported.

#### 2.6.3 Motion analysis

Head-motion-induced signal fluctuations can bias functional connectivity analyses and the resulting connectivity estimates (Satterthwaite et al., 2012, Parkes et al., 2018). In our study, this bias is especially problematic if the extent of head motion would systematically differ between patients and controls. A further potential bias could be introduced by a significant correlation between head motion and PPI. To assess these potential systematic effects, we computed framewise displacement (FD), defined as the sum of the absolute temporal derivatives of the realignment estimates (Power et al., 2012). FD was derived from the translational and rotational motion parameters obtained with SPM’s motion correction. With 304 functional volumes, each subject had 303 FD values characterizing between-volume motion. For each subject, we assessed the head motion by the mean FD, the maximum FD, and the number of frames with FD > 1 mm. Thereafter, a statistical analysis was performed to detect significant FD differences between patients and controls. Further correlation analysis tested for a significant relationship between PPI and motion (mean FD and maximum FD) across all participants, across patients, and across controls.

## 3 Results

### 3.1 Demographics and clinical scales

During MRI preprocessing, two participants (1 FMD, 1 HC) were removed from the sample due to faulty data. The final research sample size consisted of 77 participants: 38 FMD patients and 39 healthy controls. Between group comparisons were performed on basic demographic measurements and a variety of questionnaires and assessments. For a detailed comparison, see Table 1.

**Table 1:** Demographics and clinical assessment scores.

**Variable**	**FMD (n = 38)**	**HC (n = 39)**	**Test statistic**	**p-value (corrected)**
Age	40.0 (21.5)	41.0 (18.0)	U = 796	0.58
Sex, female	28 (74)	27 (69)	–	–
Education, years	13.0 (1.0)	15.0 (6.0)	U = 933	0.005**
BMI	27.6 (7.8)	24.6 (7.3)	U = 553	0.65
Antidepressant medication	20 (52.6)	3 (7.7)	–	–
Prepulse inhibition	0.4 (0.3)	0.7 (0.2)	U = 1059	0.005**
Disease duration, years	3.0 (6.0)	–	–	–
S-FMDRS total score	10.0 (6.8)	–	–	–
Beck Depression Inventory-II (BDI-II)	17.0 (17.0)	4.5 (7.5)	U = 262	<0.001***
State Anxiety Inventory	50.0 (12.0)	35.0 (10.5)	U = 217	<0.001***
Trait Anxiety Inventory	50.0 (16.2)	38.0 (14.0)	U = 238	<0.001***
Fatigue Severity Scale (FSS)	5.7 (2.5)	3.5 (2.2)	U = 256	<0.001***
Pain intensity (composite score)	5.5 (4.5)	0.8 (2.0)	U = 156	<0.001***
FPHQ	21.0 (11.0)	4.5 (4.0)	U = 32	<0.001***
FPHQ without motor symptoms	15.0 (11.8)	4.0 (4.0)	U = 68	<0.001***
SMSS	5.5 (5.0)	0.0 (0.0)	U = 22	<0.001***
Note: Data reported as median (inter-quartile range) except for sex and antidepressant medication, which are reported as frequency (percentage) Antidepressant medication defined as selective serotonin reuptake inhibitors or serotonin–norepinephrine reuptake inhibitors IQR = Q75 − Q25 (single value representing spread) Statistical tests: Mann-Whitney U tests for continuous variables Multiple comparison correction: Bonferroni-Holm method applied to all p-values Test statistic: U = Mann-Whitney U statistic FMD = Functional Movement Disorder patients; HC = Healthy Controls Significance levels based on corrected p-values: * p < 0.05, ** p < 0.01, *** p < 0.001				

### 3.2 PPI size differences

**Fig. 1:**
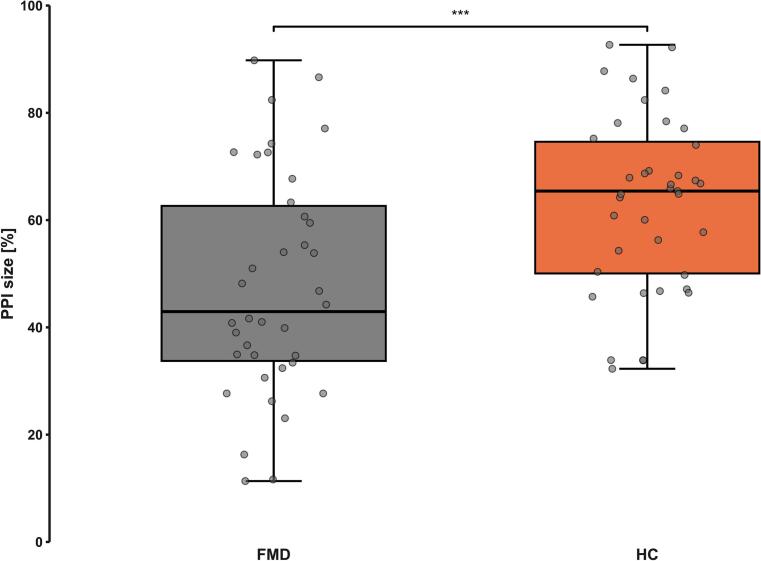
Between group comparison of prepulse inhibition (PPI) size. PPI size in patients with functional movement disorders (FMD) and healthy controls (HC). The PPI size was lower in the FMD group than in the HC. PPI size is operationalized as the difference between the mean of the blink reflex magnitude in baseline and prepulse trials. *** p < 0.001.

Prepulse inhibition size was significantly higher in healthy controls compared to FMD patients (Fig. 1). Correlation tests of PPI size with the other assessed variables were performed, with age, STAI, PAIN and FPHQ being significant. After correction for multiple comparisons, only PAIN showed a significant negative correlation, where higher pain scores were associated with lower PPI. For the complete overview, see Table 2.

**Table 2:** Spearman correlations between PPI size and clinical symptom scales.

**Variable**	**r**	**p**	**p (corrected)**	**n**
Beck Depression Inventory-II (BDI-II)	−0.183	0.12	0.22	75
State Anxiety Inventory	−0.227	0.050*	0.14	75
Fatigue Severity Scale (FSS)	−0.078	0.51	0.51	75
Pain intensity (composite score)	−0.330	0.004**	0.019*	75
FPHQ	−0.274	0.017*	0.07	75
SMSS	0.028	0.87	0.91	38
S-FMDRS total score	−0.065	0.70	0.91	38
				
r = Spearman correlation coefficient; n = number of paired observations Prepulse inhibition calculated as 1 − PPI (higher values = better inhibition) S-FMDRS and SMSS are compared for FMD group onlyPain intensity (composite score) = mean of current pain and average pain ratings Significance: * p < 0.05, ** p < 0.01, *** p < 0.001p-value (Holm-Sidak) is corrected for multiple comparisons				

### 3.3 Seed-Based connectivity analysis

Examining the main effect of group on SBC showed an increased connectivity in the FMD group from various seeds, including the left cerebellum or frontal orbital cortex. The full list of clusters is provided in Supplementary Table 1. The HC group showed an increased connectivity from the right inferior frontal gyrus seed and the occipital pole, among others. The full list is provided in Supplementary Table 2.

The SBC analysis revealed significant Group × PPI size interactions on connectivity for 5 seeds, showing a difference in the relationship between PPI size and connectivity between the FMD and HC groups. These were the left insular cortex, the left and right anterior insula, the right supramarginal gyrus (SMG), as well as the right posterior division of the SMG. The pattern of these interactions was highly consistent across seeds, with significant clusters found predominantly in temporoparietal areas including the SMG or the superior temporal gyrus (STG). A comprehensive overview of all significant interaction clusters is provided in Supplementary Fig. 1 and in Table 3.

**Table 3:** Detailed statistics for clusters showing significant Group × PPI interaction.

**Seed**	**Cluster Size (k)**	**Region**	**Hemisphere**	**P-FWE**	**P**	**T**	**x**	**y**	**z**
Insular Cortex Left	316	**Planum Temporale (25.4%)** Parietal Opercular Cortex (13.1%) Supramarginal Gyrus, posterior division (3.6%)	Left	0.015	0.002	5.79	−36	−38	14
	387	**Planum Temporale (21.8%)** Superior Temporal Gyrus, posterior division (15.2%) Parietal Opercular Cortex (4.9%)	Right	0.006	0.001	5.65	58	−18	4
SMG R	550	**Parietal Opercular Cortex (21.9%)** Planum Temporale (16.7%) Heschl's Gyrus (6.4%)	Left	0.001	0	5.24	−42	−38	14
	415	**Parietal Opercular Cortex (24.6%)** Planum Temporale (24.5%) Heschl's Gyrus (11.1%)	Right	0.004	0.001	4.65	52	−24	14
	422	**Supramarginal Gyrus, anterior division (36.4%)** Postcentral Gyrus (26.9%) Parietal Opercular Cortex (2.5%)	Left	0.004	0	4.5	−56	−26	46
pSMG r	587	**Central Opercular Cortex (14.7%)** Planum Polare (8.9%) Insular Cortex (8.4%)	Left	0.001	0	5.16	–32	−4	−4
	372	**Cingulate Gyrus, anterior division (28.3%)** Juxtapositional Lobule Cortex (11.1%) Paracingulate Gyrus (9.6%)	Bilateral	0.009	0.001	4.72	14	−6	42
	757	**Planum Temporale (22.5%)** Parietal Opercular Cortex (21.0%) Heschl's Gyrus (6.9%)	Right	0	0	4.39	48	−26	18
AInsula L	441	**Planum Temporale (19.4%)** Superior Temporal Gyrus, posterior division (6.8%) Parietal Opercular Cortex (6.4%)	Left	0.003	0	5.03	−44	−38	12
	343	**Planum Temporale (18.1%)** Superior Temporal Gyrus, posterior division (12.7%) Heschl's Gyrus (3.6%)	Right	0.009	0.001	5.02	60	−18	2
AInsula R	308	**Planum Temporale (22.3%)** Parietal Opercular Cortex (12.3%) Superior Temporal Gyrus, posterior division (9.1%)	Left	0.016	0.002	4.55	−34	−38	14
	472	**Superior Temporal Gyrus, posterior division (17.5%)** Planum Temporale (9.0%) Superior Temporal Gyrus, anterior division (5.2%)	Right	0.002	0	4.49	44	−20	−4

To illustrate the most consistent findings, we will detail the results from the two seeds that yielded the highest T-values: the left insular cortex and the right supramarginal gyrus.

#### 3.3.1 Left insular Cortex

**Fig. 2:**
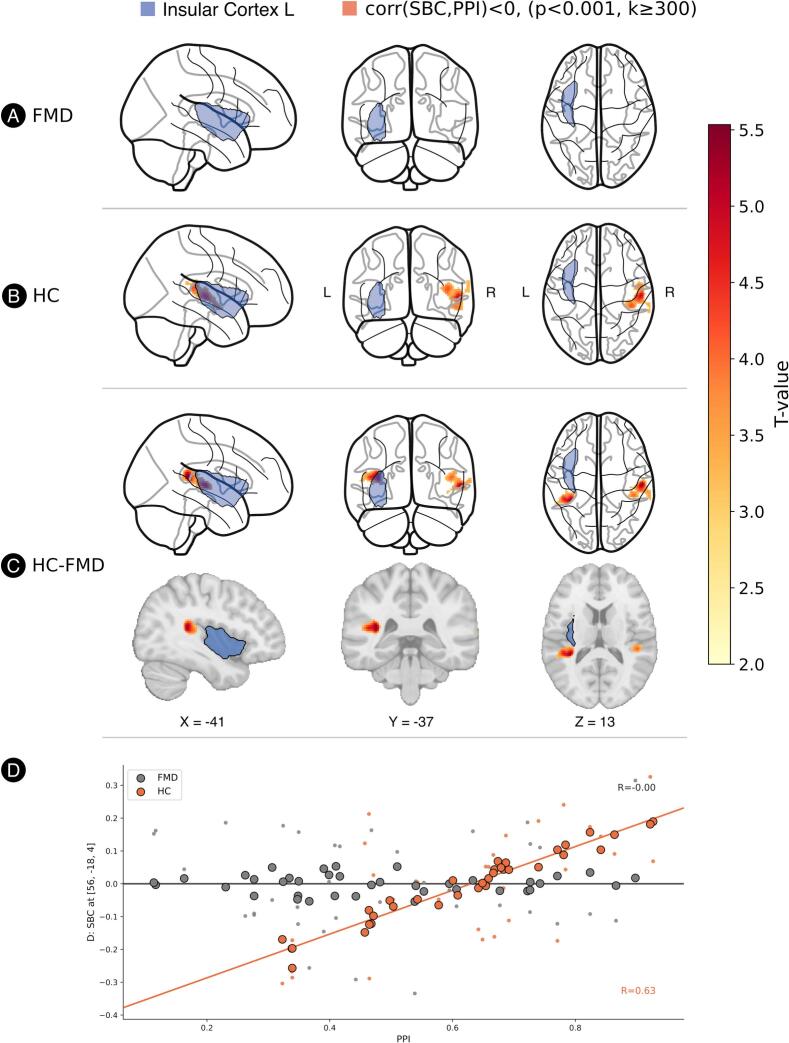
SBC results of the left insular cortex seed. Positive association between seed‑based connectivity (SBC) from the left insular cortex and prepulse inhibition (PPI) in healthy controls (HC). (A) In functional movement disorder (FMD) patients, no significant correlation was found. (B) In HC, a significant positive correlation between PPI and SBC was identified in the right temporoparietal junction (TPJ) cluster. Results are shown at a voxel‑level threshold of *p* < 0.001 and cluster‑extent threshold *k* ≥ 300 voxels (family‑wise error–corrected at the cluster level). (C) A significant group × PPI interaction in bilateral TPJ regions meets the same statistical criteria (*p* < 0.001, *k* ≥ 300, cluster-level corrected). Seed region is shown in blue; significant TPJ clusters are displayed in yellow‑through‑red, with color intensity indicating t‑value strength. (D) Scatterplot of SBC extracted at the global‑maximum (MNI: [56, −18, 4]). Large dots represent fitted model values; small dots represent mean‑centered raw SBC values. Data are plotted separately for FMD (grey) and HC (orange). (For interpretation of the references to colour in this figure legend, the reader is referred to the web version of this article.)

The analysis revealed a significant Group × PPI size interaction in two temporoparietal clusters extending from the left and right planum temporale into the left SMG and right STG respectively. In the right cluster, this interaction was characterized by a significant positive correlation between PPI size and connectivity in the healthy control group (See Fig. 2 for a graphical overview). In contrast, no significant relationship was observed in the FMD group. Furthermore, there was no significant main effect of the group on mean connectivity in this seed.

#### 3.3.2 Right supramarginal gyrus

**Fig. 3:**
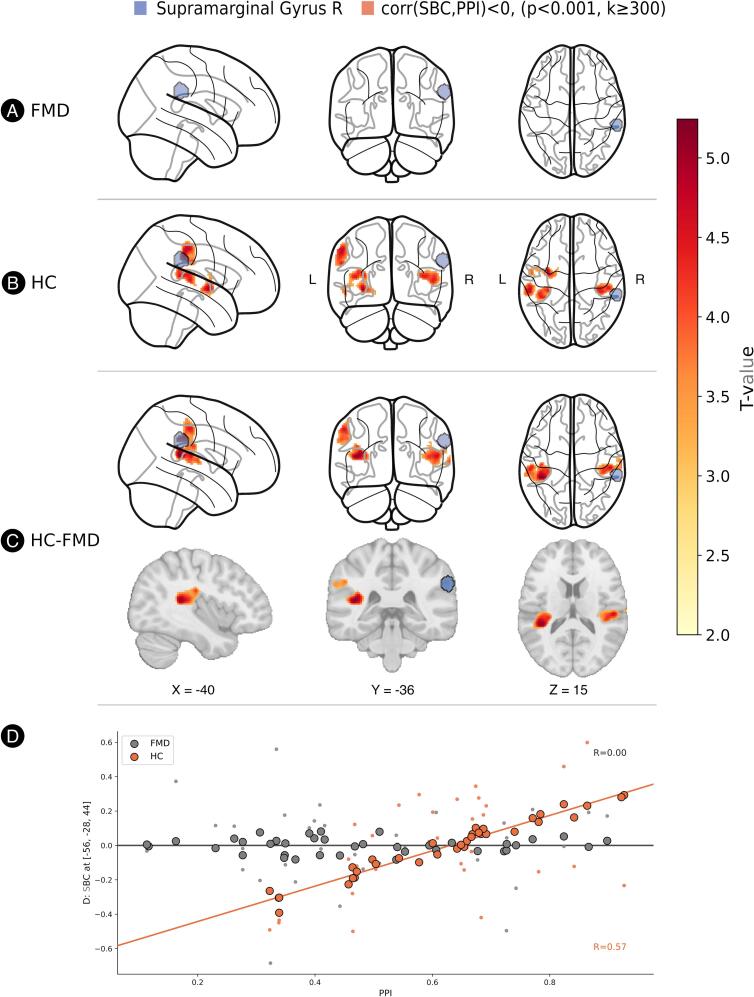
SBC results of the right supramarginal gyrus seed. Positive association between seed‑based connectivity (SBC) from the right supramarginal gyrus and prepulse inhibition (PPI) in healthy controls (HC). (A) No significant PPI–SBC correlation was observed in functional movement disorder (FMD) patients. (B) In HCs, a significant positive PPI versus SBC correlation was identified in bilateral temporoparietal junction (TPJ) clusters. Statistical thresholds applied: voxel‑level *p* < 0.001 and cluster‑extent *k* ≥ 300 voxels (cluster‑level FWE correction). (C) A significant group × PPI interaction was detected in the same bilateral TPJ regions under identical statistical thresholds (*p* < 0.001, *k* ≥ 300, cluster‑level corrected). Seed region is shown in blue; significant clusters in yellow/red color scale, indicating t‑value strength. (D) Scatterplot of SBC extracted at the global maximum (MNI: [‑56, ‑28, 44]). Large dots represent fitted model values; small dots represent mean‑centered raw SBC values. Data are plotted separately for FMD (grey) and HC (orange). (For interpretation of the references to colour in this figure legend, the reader is referred to the web version of this article.)

A similar pattern was observed for the right SMG seed. A significant Group × PPI size interaction was found in three temporoparietal clusters, which included the left and right planum temporale and the left SMG. Again, post-hoc tests confirmed this was driven by a significant positive correlation between PPI size and connectivity in healthy controls, a relationship that was absent in FMD patients (See Fig. 3 for a graphical overview). There was no significant main effect of the group on mean connectivity in this seed.

### 3.4 Motion analysis

The analysis of head motion during MRI scanning revealed only subtle effects. For all participants, mean FD was below 0.7 mm. Across the entire study (77 participants × 304 image volumes; 23,408 frames), only 268 frames showed single-frame movements > 1 mm (1.1%). For both mean FD and maximum FD, statistical analysis did not reveal any significant head motion differences between patients and controls (*P* > 0.2). Using mean FD and maximum FD, we also did not find any significant correlation between head motion and PPI across all participants, within patients, and within controls (*P* > 0.3).

## 4 Discussion

In this study, we investigated whether individual differences in PPI of the blink reflex – a robust neurophysiological measure of sensory gating at the subcortical level – were associated with functional connectivity alterations in patients with FMD. Consistent with our previous work (Hanzlíková et al., 2019, Nováková et al., 2025), individuals with FMD showed impaired PPI and association with self-reported measures of pain. We found differences in mean connectivity between the two groups across various seeds including the cerebellum, frontal orbital cortex or the inferior frontal gyrus.

The relationship between PPI and resting-state connectivity differed between FMD patients and HCs, most consistently for the left insula and right supramarginal gyrus seeds. Healthy controls exhibited a robust positive correlation between PPI size and connectivity from the bilateral insula to the left SMG, right STG and bilateral planum temporale. These areas, especially SMG and STG are considered to be core components of the temporoparietal junction (TPJ), according to previous research (Bzdok et al., 2013). The same relationship was found between the left and the right TPJ in HCs, but was absent in patients with FMD. These findings indicate that in healthy individuals, higher PPI size is linked to stronger functional coupling between the insula and TPJ as well as between the left and right TPJ. This coupling appears to be disrupted in individuals with FMD. The absence of the normal PPI-connectivity relationship in FMD patients may indicate a failure of top-down regulation of the brain to appropriately filter sensory information based on attentional and salience demands.

Extensive research has identified the neuroanatomical and neurochemical substrates of PPI. While the core circuitry resides in the brainstem (Koch, 1999, Valls-Sole, 2012), animal models have demonstrated robust top-down modulation of PPI by cortico-limbic-striatal structures (Swerdlow and Geyer, 1998, Swerdlow et al., 2001). Our findings implicating the left insula and right supramarginal gyrus in PPI regulation align with existing evidence from humans, where neuroimaging studies support the recruitment of analogous cortico-striato-pallido-thalamic circuitry during PPI (Naysmith, Kumari and Williams, 2021) and modulation of PPI by a distributed network including both subcortical regions (thalamus, striatum, amygdala, hippocampus) and cortical areas (insula, anterior cingulate cortex, inferior frontal gyrus, and inferior parietal lobule/supramarginal gyrus), highlighting the contribution of higher-order regions and top-down influences on sensory gating (Kumari et al., 2005, Campbell et al., 2007, Cano et al., 2021). Notably, in studies utilizing somatosensory prepulse, the involvement of the insula as well as the areas of the TPJ (inferior parietal lobule or supramarginal gyrus) is a repeated finding (Kumari et al., 2003, Kumari et al., 2007, Kumari et al., 2008, Zebardast et al., 2013). Higher activation in these areas during PPI was demonstrated in healthy controls, while it was reduced in schizophrenia patients (Kumari et al., 2003, Kumari et al., 2007), men with higher psychosis-proneness (Kumari, Antonova and Geyer, 2008) as well as in adults with Tourette Syndrome (Zebardast et al., 2013).

TPJ has been associated with disparate processes including sense of agency (Zito et al., 2020), theory of mind (Saxe and Kanwisher, 2003) and multisensory integration (Matsuhashi et al., 2004). The right TPJ, together with the anterior insula, forms a core node of the ventral attentional network (VAN) (Fox et al., 2006, Farrant and Uddin, 2015), a system crucial for detecting and filtering salient stimuli. Within this network, the insula plays a pivotal role by coordinating attentional shifts and evaluating the salience of incoming sensory information (Menon and Uddin, 2010). As an automatic, pre-conscious sensory selection, PPI size is influenced by stimulus salience (Filion et al., 1993, Corbetta et al., 2008, Sara and Bouret, 2012, Correa et al., 2019, Kofler et al., 2024). An impaired PPI can interfere with the appropriate integration or filtering of low-salience inputs against high-salience inputs, leading to decreased sensory fidelity. Against this background, the observed relationship between PPI and insula-TPJ connectivity possibly reflects the role of VAN in integrating multisensory information and modulating sensory processing through top-down attentional mechanisms. Our finding of absent PPI-connectivity coupling in FMD aligns with previous evidence of aberrant insula-TPJ connectivity in FMD, which has been reported in both static connectivity analyses (Maurer et al., 2016) and dynamic co-activation approaches (Weber et al., 2025). Reduced functional connectivity between TPJ and insula has also been demonstrated in other psychiatric conditions such as schizophrenia, where connectivity within these circuits correlates with symptom severity (Penner et al., 2018) and where abnormal PPI is a well-established feature (Allen et al., 2009), further supporting the clinical relevance of this circuit in PPI modulation.

Additionally, we found a positive association between PPI size and connectivity from right to left TPJ (clusters in the left SMG and other surrounding areas of the inferior parietal lobule) observed in healthy controls but absent in FMD patients. Interhemispheric connectivity between TPJ regions is well-established in healthy populations (Igelström, Webb and Graziano, 2015). Disrupted interhemispheric connectivity has been documented across multiple psychiatric and neurological disorders (Yao and Kendrick, 2022). A resting-state fMRI study of somatization disorder reported significantly reduced connectivity in the SMG, as well as the angular gyrus and insula, in patients compared to controls (Su et al., 2016). Similar patterns of reduced connectivity in the inferior parietal lobule have been reported in major depressive disorder (Liu et al., 2021) and schizophrenia (Guo et al., 2018), the latter of which shows consistently reduced prepulse inhibition. This pattern suggests that effective sensorimotor gating may require not only unilateral network integrity but also coordinated bilateral processing.

The predictive coding framework – an influential account of FND – is a computational theory proposing that the brain operates as a hierarchical inference system, continuously generating predictions about incoming sensory information and comparing them against actual sensory input (Keller and Mrsic-Flogel, 2018). Central to this framework are two key concepts: prediction errors, which represent the mismatch between predicted and actual sensory signals, and precision weighting, which determines the relative gain or reliability assigned to these prediction errors based on contextual factors (Keller and Mrsic-Flogel, 2018, Haarsma et al., 2021). In FND, it has been proposed that symptoms arise from aberrant precision weighting of sensory predictions and prediction errors (Edwards et al., 2012). Although PPI reflects an automatic, largely brainstem-mediated gating mechanism, it is sensitive to top-down modulation from cortical salience and attentional systems (Swerdlow, Braff and Geyer, 2016). Within this framework, such modulation can be understood as the contextual adjustment of precision applied to sensory signals, including the prepulse stimulus (Edwards et al., 2012, Keller and Mrsic-Flogel, 2018). Disrupted insula–TPJ connectivity in FMD may therefore indicate disrupted precision control over incoming sensory cues, including the prepulse, which normally serves as a reliable signal for attenuating the subsequent startle response. A failure to assign appropriate precision to the prepulse would limit its influence on downstream gating circuits, offering a higher-level explanatory account that is compatible with established PPI neurobiology. This interpretation situates the absent PPI–connectivity coupling in FMD within broader models proposing that abnormalities in salience attribution and precision weighting contribute to the sensorimotor processing abnormalities characteristic of FMD (Sadnicka et al., 2020, Stoffel et al., 2025).

In line with our previous studies we found decreased PPI in patients with FMD (Hanzlíková et al., 2019). We also found an association of lower PPI with higher experienced pain intensity, consistent with our recent study demonstrating association between PPI and pain widespreadness and other subjective symptom severity (Nováková et al., 2025). Additionally, we found an association with subjective symptom severity in the present study, although it has not survived correction for multiple comparisons. Objective motor symptom severity was not related to PPI size in any of the studies. Establishing a mechanistic link between the experience of different symptoms and an objective neurophysiological marker is important for understanding FMD pathophysiology. PPI could be a promising biomarker for experienced symptoms, such as pain, and the mapping of PPI to neural circuits can also have therapeutic implications. The right TPJ, implicated in our study, has previously been linked to an abnormal sense of agency in patients with FND (Edwards et al., 2011, Nahab et al., 2017, Weber et al., 2025). A recent study has examined the effects of repetitive transcranial magnetic stimulation (rTMS) modulation of the rTPJ in FND patients, which led to an increase in rTPJ activity in patients, but not in controls (Bühler et al., 2024). However, the study found no significant effects on subjective judgement of agency. A small sample pilot of study of rTMS modulation of rTPJ in patients with psychogenic non-epileptic seizures (PNES) found a significant decrease of weekly seizure frequency lasting 3 months later (Peterson et al., 2018). Another study on patients with functional motor symptoms used transcranial direct-current stimulation (tDCS) targeting the right posterior parietal cortex and found a significantly higher interoceptive sensitivity after real tDCS compared to sham stimulation (Demartini et al., 2019). These findings further support the role of rTPJ in functional disorders and suggest its potential for modulation in treatment, although the findings remain limited, and vary based on methodology and FND phenotype. Our findings suggest that non-invasive brain stimulation of the identified regions, either as a standalone intervention or combined with psychotherapy, warrants evaluation in future trials for its potential to improve non-motor symptoms.

Several limitations should be considered when interpreting these findings. First, the definition of TPJ is not anatomically uniform across studies, and our analyses used regions such as the supramarginal gyrus from the atlases included in CONN toolbox as proxies for the TPJ. Future work using more refined parcellations or task-based localization could provide greater specificity. Second, the prepulse inhibition measure was obtained outside of the MRI session, which limits inferences about direct neural coupling during sensorimotor gating. Simultaneous acquisition of PPI and fMRI would enable stronger mechanistic conclusions. Future studies should also explore additional imaging modalities beyond rs-fMRI, such as diffusion tensor imaging. Third, the spatial resolution of resting-state fMRI limits the sensitivity to brainstem structures that are central to the startle circuit, preventing assessment of their connectivity with cortical regions. Fourth, although our sample size (n = 77) is comparable to prior FMD neuroimaging studies, it may limit power to detect subtle interaction effects. Several factors support the robustness of our findings: (1) we employed stringent statistical correction (cluster-level FWE and Bonferroni correction for multiple seeds); (2) the Group × PPI interactions emerged consistently across five independent seeds, converging on bilateral TPJ. Nevertheless, replication in larger independent samples is warranted. Finally, the cross-sectional design precludes conclusions about causality or the directionality between altered connectivity and impaired sensory gating. Longitudinal and interventional studies, particularly those integrating clinical outcomes and symptom modulation, will be critical to determine whether insula–TPJ network dysfunction represents a stable trait or a reversible marker of FMD pathophysiology.

## 5 Conclusions

In summary, this study provides the first evidence linking impaired sensorimotor gating, indexed by reduced prepulse inhibition, to disrupted functional connectivity between the insula and temporoparietal junction in patients with functional movement disorder. In healthy individuals, stronger connectivity within this insula–TPJ circuit, as well as between bilateral TPJ regions, was associated with more effective sensorimotor gating, suggesting that intact top-down modulation of sensory processing relies on coordinated activity within salience and ventral attention networks. The absence of this relationship in FMD points to a breakdown in the neural systems that assign salience and integrate sensory signals across modalities and hemispheres, which are crucial mechanisms for adaptive filtering of sensory information. These findings bridge neurophysiological and neuroimaging evidence of sensory processing abnormalities in FND and support predictive coding models proposing that functional symptoms emerge from aberrant precision weighting of sensory predictions.

## CRediT authorship contribution statement

**David Voženílek:** Writing – original draft, Visualization, Formal analysis. **Karsten Mueller:** Writing – review & editing, Supervision, Formal analysis. **Tereza Serranová:** Writing – review & editing, Supervision, Resources, Methodology, Conceptualization. **Lucia Nováková:** Resources, Investigation. **Manuela Vaněčková:** Writing – review & editing, Resources. **Petr Sojka:** Writing – review & editing, Visualization, Supervision.

## Funding

This work was supported by the Czech Ministry of Health, project AZV NW24-04–00456 and the General University Hospital in Prague project MH CZ-DRO-VFN64165.

## Declaration of competing interest

The authors declare that they have no known competing financial interests or personal relationships that could have appeared to influence the work reported in this paper.

## Data Availability

Data will be made available on request.
