# Supplementary figures and images for: Altered functional connectivity related to prepulse inhibition in functional movement disorder
Source: Neuroimage Clin. 2026 Feb 15;49:103966. doi: 10.1016/j.nicl.2026.103966 (PMC12926577; doi:10.1016/j.nicl.2026.103966)

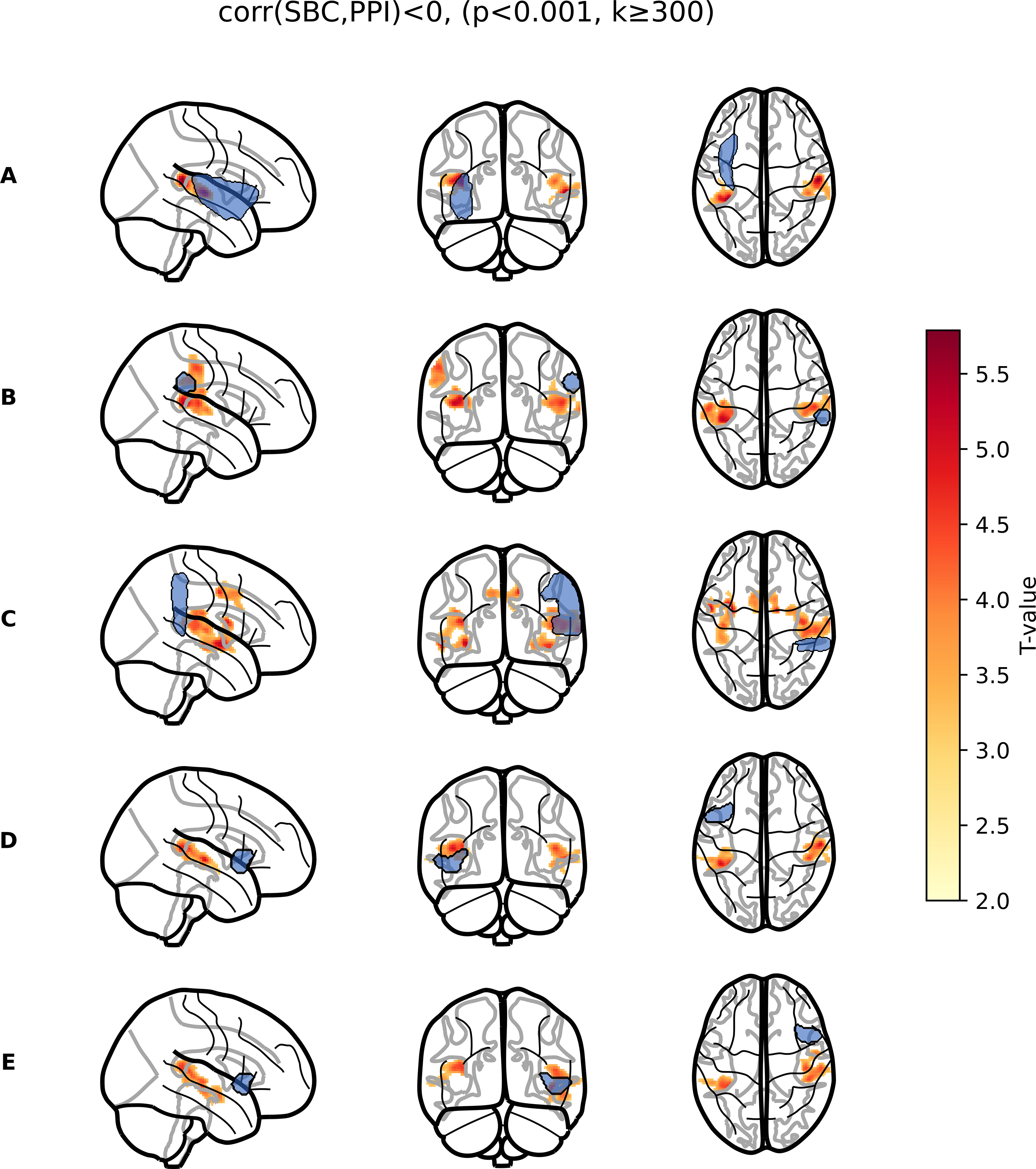

Supplement: Supplementary Fig. 1 [file mmc3.jpg]
