# Supplementary material for: Altered functional connectivity related to prepulse inhibition in functional movement disorder
Source: Neuroimage Clin. 2026 Feb 15;49:103966. doi: 10.1016/j.nicl.2026.103966 (PMC12926577; doi:10.1016/j.nicl.2026.103966)
Supplement: Supplementary Data 1 [file mmc1.docx]

**Supplementary table 1.** Regions exhibiting increased connectivity in patients with FMD compared to healthy controls (FMD > HC).

| **Seed** | **Cluster Size (k)** | **P-FWE** | **P** | **T** | **x** | **y** | **z** |
| --- | --- | --- | --- | --- | --- | --- | --- |
| Cerebelum 7b Left | 372 | 0,01 | 0,001 | 6,04 | 32 | -20 | 16 |
| Cerebelum 7b Left | 322 | 0,019 | 0,003 | 4,19 | -42 | -24 | 18 |
| Frontal Orbital Cortex Left | 844 | 0 | 0 | 5,82 | 20 | -60 | 44 |
| Frontal Orbital Cortex Left | 1018 | 0 | 0 | 5,07 | 52 | -28 | 36 |
| Frontal Orbital Cortex Left | 1404 | 0 | 0 | 4,64 | -52 | -30 | 32 |
| Frontal Operculum Cortex Left | 309 | 0,016 | 0,002 | 5,69 | 32 | -56 | -24 |
| Frontal Operculum Cortex Left | 546 | 0,001 | 0 | 4,79 | -16 | -66 | 52 |
| Frontal Operculum Cortex Left | 436 | 0,003 | 0 | 4,73 | -46 | -32 | 36 |
| Cingulate Gyrus, anterior division | 656 | 0 | 0 | 5,68 | 40 | -80 | 30 |
| Cingulate Gyrus, anterior division | 440 | 0,004 | 0 | 4,82 | -34 | -40 | -18 |
| Cingulate Gyrus, anterior division | 538 | 0,001 | 0 | 4,67 | -20 | -74 | 56 |
| Cingulate Gyrus, anterior division | 480 | 0,002 | 0 | 4,66 | 38 | -48 | -42 |
| Inferior Frontal Gyrus Right | 779 | 0 | 0 | 5,6 | 18 | -60 | 46 |
| Inferior Frontal Gyrus Right | 378 | 0,008 | 0,001 | 5,14 | 24 | -16 | 8 |
| Inferior Frontal Gyrus Right | 404 | 0,006 | 0,001 | 4,27 | -36 | 24 | 6 |
| Cerebelum 4 5 Left | 389 | 0,008 | 0,001 | 5,56 | -46 | -50 | 32 |
| Cerebelum 4 5 Left | 589 | 0,001 | 0 | 5,05 | -10 | -60 | 46 |
| Caudate Right | 359 | 0,011 | 0,001 | 5,07 | 10 | -68 | -20 |
| Caudate Right | 375 | 0,009 | 0,001 | 4,53 | -40 | -24 | 36 |
| Superior Parietal Lobule Left | 331 | 0,016 | 0,002 | 5,05 | -18 | 16 | 0 |
| Superior Parietal Lobule Left | 397 | 0,007 | 0,001 | 3,86 | -6 | 46 | 8 |
| Angular Gyrus Right | 374 | 0,008 | 0,001 | 4,93 | 24 | -36 | -22 |
| Angular Gyrus Right | 335 | 0,013 | 0,002 | 4,81 | -14 | -22 | 62 |
| Superior Temporal Gyrus, posterior division Left | 593 | 0,001 | 0 | 4,89 | 48 | -66 | 20 |
| Superior Temporal Gyrus, posterior division Left | 393 | 0,008 | 0,001 | 4,63 | 18 | -60 | 50 |
| Superior Temporal Gyrus, posterior division Left | 389 | 0,008 | 0,001 | 4,09 | -52 | -26 | 30 |
| Middle Temporal Gyrus, temporooccipital part Right | 479 | 0,004 | 0,001 | 4,83 | -14 | -46 | -18 |
| Middle Temporal Gyrus, temporooccipital part Right | 754 | 0 | 0 | 4,82 | -20 | -4 | 4 |
| Middle Temporal Gyrus, temporooccipital part Right | 505 | 0,003 | 0 | 4,29 | 22 | -52 | 64 |
| Middle Temporal Gyrus, temporooccipital part Right | 383 | 0,012 | 0,002 | 4,01 | -2 | -10 | 68 |
| Superior Temporal Gyrus, posterior division Right | 317 | 0,021 | 0,003 | 4,67 | -24 | -12 | 12 |
| Superior Temporal Gyrus, posterior division Right | 397 | 0,008 | 0,001 | 4,12 | 20 | -52 | 64 |
| Temporal Occipital Fusiform Cortex Left | 310 | 0,021 | 0,003 | 4,66 | -46 | -42 | 24 |
| Temporal Occipital Fusiform Cortex Left | 455 | 0,004 | 0 | 4,55 | 18 | 54 | 0 |
| Cerebelum 4 5 Right | 967 | 0 | 0 | 4,65 | -16 | -62 | 30 |
| Cerebelum 4 5 Right | 301 | 0,025 | 0,003 | 4,18 | -42 | -50 | 28 |
| Lateral Occipital Cortex, superior division Right | 643 | 0,001 | 0 | 4,62 | -2 | 32 | 14 |
| Lateral Occipital Cortex, superior division Right | 376 | 0,01 | 0,001 | 4,54 | 58 | -30 | -8 |
| Caudate Left | 640 | 0,001 | 0 | 4,56 | -44 | -28 | 34 |
| Caudate Left | 711 | 0 | 0 | 4,45 | -28 | -48 | 52 |
| Caudate Left | 327 | 0,017 | 0,002 | 4,39 | 42 | -72 | 14 |
| Caudate Left | 516 | 0,002 | 0 | 4,14 | 64 | -36 | 24 |
| Caudate Left | 558 | 0,001 | 0 | 3,99 | 24 | -32 | 48 |
| Middle Frontal Gyrus Left | 368 | 0,009 | 0,001 | 4,56 | -38 | -32 | 60 |
| Middle Frontal Gyrus Left | 388 | 0,007 | 0,001 | 4,3 | -18 | -24 | 56 |
| Temporal Occipital Fusiform Cortex Right | 718 | 0 | 0 | 4,32 | 6 | 38 | 4 |
| Temporal Occipital Fusiform Cortex Right | 317 | 0,016 | 0,002 | 4,16 | -54 | -46 | 20 |

**Supplementary table 2.** Regions exhibiting increased connectivity in healthy controls compared to patients with FMD (HC > FMD).

| **Seed** | **Cluster Size (k)** | **P-FWE** | **P** | **T** | **x** | **y** | **z** |
| --- | --- | --- | --- | --- | --- | --- | --- |
| Inferior Frontal Gyrus, pars triangularis Right | 813 | 0 | 0 | 5,83 | 18 | 26 | 16 |
| Inferior Frontal Gyrus, pars triangularis Right | 402 | 0,006 | 0,001 | 4,9 | -24 | 18 | 30 |
| Occipital Pole Right | 629 | 0,001 | 0 | 5,46 | -28 | -28 | 62 |
| Occipital Pole Right | 425 | 0,006 | 0,001 | 4,61 | 36 | -24 | 58 |
| Inferior Frontal Gyrus Right | 757 | 0 | 0 | 5,29 | 28 | 0 | 30 |
| Inferior Frontal Gyrus Right | 612 | 0,001 | 0 | 5,18 | 6 | 36 | 48 |
| Inferior Frontal Gyrus, pars triangularis Left | 429 | 0,004 | 0 | 4,9 | -16 | 12 | 24 |
| Inferior Frontal Gyrus, pars triangularis Left | 338 | 0,012 | 0,001 | 4,5 | 26 | -34 | 18 |
| Inferior Frontal Gyrus, pars opercularis Left | 337 | 0,016 | 0,002 | 4,66 | 24 | 40 | 6 |
| Inferior Frontal Gyrus, pars opercularis Left | 420 | 0,006 | 0,001 | 4,57 | 24 | -24 | 30 |
