# Supplementary material for: Altered functional connectivity related to prepulse inhibition in functional movement disorder
Source: Neuroimage Clin. 2026 Feb 15;49:103966. doi: 10.1016/j.nicl.2026.103966 (PMC12926577; doi:10.1016/j.nicl.2026.103966)
Supplement: Supplementary Data 2 [file mmc2.docx]

**Supplementary table 2.** Regions exhibiting increased connectivity in healthy controls compared to patients with FMD (HC > FMD).

| **Seed** | **Cluster Size (k)** | **P-FWE** | **P** | **T** | **x** | **y** | **z** |
| --- | --- | --- | --- | --- | --- | --- | --- |
| Inferior Frontal Gyrus, pars triangularis Right | 813 | 0 | 0 | 5,83 | 18 | 26 | 16 |
| Inferior Frontal Gyrus, pars triangularis Right | 402 | 0,006 | 0,001 | 4,9 | -24 | 18 | 30 |
| Occipital Pole Right | 629 | 0,001 | 0 | 5,46 | -28 | -28 | 62 |
| Occipital Pole Right | 425 | 0,006 | 0,001 | 4,61 | 36 | -24 | 58 |
| Inferior Frontal Gyrus Right | 757 | 0 | 0 | 5,29 | 28 | 0 | 30 |
| Inferior Frontal Gyrus Right | 612 | 0,001 | 0 | 5,18 | 6 | 36 | 48 |
| Inferior Frontal Gyrus, pars triangularis Left | 429 | 0,004 | 0 | 4,9 | -16 | 12 | 24 |
| Inferior Frontal Gyrus, pars triangularis Left | 338 | 0,012 | 0,001 | 4,5 | 26 | -34 | 18 |
| Inferior Frontal Gyrus, pars opercularis Left | 337 | 0,016 | 0,002 | 4,66 | 24 | 40 | 6 |
| Inferior Frontal Gyrus, pars opercularis Left | 420 | 0,006 | 0,001 | 4,57 | 24 | -24 | 30 |
